# Selected Tools for Assessing the Risk of Falls in Older Women

**DOI:** 10.1155/2020/2065201

**Published:** 2020-11-12

**Authors:** Patrycja Bobowik, Ida Wiszomirska, Anna Leś, Katarzyna Kaczmarczyk

**Affiliations:** ^1^Faculty of Rehabilitation, Józef Piłsudski University of Physical Education, Warsaw, Poland; ^2^Faculty of Physical Education, Józef Piłsudski University of Physical Education, Warsaw, Poland

## Abstract

**Methods:**

Fifty-five females were examined (May 2018-June 2019). Stabilographic examinations were performed with eyes open (EO) and closed (EC). An analysis of variance (ANOVA) and Spearman rank correlation were performed to determine the relationships and differences between the above tests.

**Results:**

The results of the TUG correlate with the overall stability index (OSI) EO (*r* = 0.314), medial-lateral stability index (MLSI) EO (*r* = 0.297), and fall risk index (FRI6-2; *r* = 0.435) in stabilographic examinations and the FRT (*r* = −0.399). The results of the modified Unterberger test correlate with MLSI EO (*r* = 0.276), OSI EC (*r* = 0.310), and MLSI EC (*r* = 0.378). There are statistically significant differences between faller and nonfaller groups in TUG (*p* = 0.0068), FRT (*p* = 0.001), and MLSI EO (*p* = 0.0118).

**Conclusions:**

The modified Unterberger test and TUG can be considered effective in functional FR assessment in older women. Using at least two different functional tests may improve the assessment of FR.

## 1. Introduction

The World Health Organization (WHO) states that between 2015 and 2050, the number of people over the age of 60 will increase from 12 to 22% [1]. One-third of older people experience a fall each year, and some of these falls could be caused by balance disorders. The rest may occur due to environmental factors or low levels of physical activity [2]. Previous studies state that 3% of all hospitalised people have suffered a fall, and 25% are injurious [3, 4]. Fall risk (FR) is still one of the most serious problems, mainly among people aged 65 and over [2, 5].

Falls are a consequence of many factors: degenerative changes in the vestibular and visual systems, deterioration of motor control, reduced muscle strength, the side effects of taking medication, and environmental factors [5, 6]. Motor functions and proprioception worsen with age [7]. Ageing also causes damage to receptors, neural pathways, and centres responsible for cognitive and equivalent functions. As a consequence, there is an abnormal flow of information between the central and peripheral nervous system [4].

Identifying the risk of falls (FR) in older people is a major challenge in daily physiotherapeutic practice, despite the availability of different types of FR assessment tools. FR can be assessed functionally or stabilographically. Usually, physiotherapists exam the balance control system and (the directly related to FR) the level of physical activity, with functional tests [8]. The relationship between FR and physical functionality has therefore been evaluated [9]. Unfortunately, the tests are not able to differentiate intrinsic factors related to falls. However, these tests are focused on functional limitations in balance or gait and provide a lot of information about the patient's functional capabilities and limitations. Luckily, their use does not incur significant costs [8, 10].

The Functional Reach Test (FRT) is a clinical measure of balance used to predict FR. The FRT evaluates anterior-posterior stability by measuring the maximum forward deflection from a neutral position and the displacement of the centre of gravity [11]. It seems that the movement of the trunk influences the test more than any displacement of the centre of pressure [12]. Notwithstanding various concerns, the FRT has been used in older people [13] and people with Parkinson's disease [11].

The Fullerton Functional Fitness Test consists of 6 elements that assess coordination, strength, agility, and balance. The Timed Up and Go (TUG) test is used more often than the other elements of the Fullerton Test [4]. It is believed that the TUG is potentially a useful tool to assess changes in a variety of substrates that affect the equivalent capabilities. It has been used to assess, for example, the physical efficiency and balance of healthy older people [4] and residents of social housing [14].

The modified Unterberger test (dynamic Romberg test), first described in 1846, is also used to assess FR. It involves walking in place with the eyes closed. It evaluates forward tilt and body rotation dynamics [15]. The reliability of the Romberg test for evaluating postural performance and determining sensory preference in postural control—at least in healthy controls—is uncertain [16]. The modified Unterberger test has not yet been evaluated for reliability. This test has however been used, for instance, in people with hearing impairments [17] and older people [19].

Body balance disorders are also diagnosed with the use of stabilographic platforms. These allow for the measurement of balance from a biomechanical perspective. Perraca et al. conclude that Biodex Balance System (BBS) SD (USA) platform measures are reliable and may be useful for measuring FR and for monitoring programs to prevent falls in older people [19]. Furthermore, stabilographic and functional tests have been used to measure the efficacy of different training regimes [4, 15]. The BBS has been used to assess balance in orthopaedic patients [18], neurological patients (Parkinson's disease) [7], people with multiple sclerosis [6], and women with hearing impairments [20]. The platform is also used to evaluate the effectiveness of functional tests [6].

The complexity of the balance control system makes assessment challenging. Previous studies have shown that functional tests' performance raises serious doubts regarding their objectivity and reliability. Sometimes, participants who get negative results in the functional test actually have balance disorders. Therefore, the utility of functional tests in clinical practice remains uncertain. This study is therefore aimed at comparing the results of the FRT, TUG, and modified Unterberger test, with the most objective tool (BBS) in the FR assessment of older women. The main question is whether the above functional tests, the most frequently used in everyday physiotherapeutic practice, are helpful and precise. Finally, in the absence of any platform, could balance disorders be diagnosed in a cheaper and more convenient way? Should FR assessment take place in a dynamic environment or in a static one?

## 2. Materials and Methods

The research received approval from the Warsaw Bioethical Commission in Poland. Selecting participants took place in two stages. The first stage involved an interview conducted by a physiotherapist. To qualify, participants had to meet multiple criteria. They had to be 65 or older, be in good mental condition, and have a low level of physical activity, as declared by them. They also had to be willing to participate in the research. The second stage consisted of another interview, a cardiac examination, and electrocardiography carried out by a physician. Exclusion criteria included are as follows: a serious mental condition, communication problems, medications that might cause imbalance, diseases that cause imbalance (e.g., Parkinson's disease), multiple sclerosis, labyrinth diseases, advanced coronary disease, and a high cardiac risk. The second stage took place in the laboratory. Two participants withdrew because of deteriorating health and sudden injury. The experiment lasted from May 2018 to June 2019. All participants provided written consent.

The research outcomes included anthropometric measurements (body height, body weight, and waist and hip circumferences). Patients' body mass index (BMI) and waist-hip ratio (WHR) were also calculated.

The FRT, TUG, and the modified Unterberger test were used to assess balance disorders. These tests are commonly used by physiotherapists in Poland to assess FR in older people. The FRT was performed in a standing position, with the feet spaced out at the width of the hips. One upper limb was raised forward until it was level with the shoulder. The participants were asked to move to the position of maximum forward tilt. The distance between the middle finger of the raised limb in the starting position and the position at maximum tilt was measured. The TUG required participant's to get out of a chair, walk a distance of 2.44 m, go round a cone, return to the chair, and get back into the starting position. The time taken was measured to the millisecond. The Unterberger test was slightly modified. It consisted of walking in place with eyes closed and upper limbs raised forward until they were level with the shoulders. Each participant was asked to raise their lower limbs to 45 degrees of flexion in the knee joints and 90 degrees in the hip joints for 20 seconds during the walk. The test was assessed as being positive if the participant moved 21 cm to the front or side while marching.

Participants were divided into two groups according to their history of falls over the preceding year. Participants with fall incidents in the preceding year formed a “fallers group,” and participants without a fall incident formed a “non-fallers group.” Due to the fact that some of the falls may have occurred due to an uncontrollable environmental factor (extrinsic risk factor), participants were divided into two groups according to the modified Unterberger test results. The test was considered positive if a participant moved more than 21 cm forward or sideways while marching (group 1). Participants with negative results (movement of less than 21 cm) formed group 2.

Stabilographic tests were performed to assess postural stability. Three protocols were prepared on the Biodex Balance System SD platform from Biodex (BBS). Each of them lasted 20 seconds. The BBS enables subjects to be tested on a stable or unstable platform on 12 levels. The degree of instability of the platform increases from 12 to 1 (the most stable platform being level 12). A Postural Stability Test (PST) was performed on a stationary platform with eyes open (EO) and closed (EC) to determine: an OSI (overall stability index), APSI (anterior-posterior stability index) and MLSI (medial-lateral stability index). High values of these indices comprised the FR. The Fall Risk Test was also carried out with EO on an unstable platform at levels ranging from 6 to 2. On this basis, the fall risk index (FRI) was determined.

The recorded data were analysed with the use of STATISTICA (v. 13) StatSoft USA. The normality of distribution was analysed using the Shapiro-Wilk test. In some cases, the results were subjected to a natural logarithmising procedure to obtain normal distribution. Each parameter was described using descriptive statistics (means and standard deviations). In addition, Levene's homogeneity of variance test was performed. The analysis of variance (ANOVA) was performed for groups of fallers and nonfallers, as well as those with positive and negative Unterberger test results. The ANOVA was performed with the stability parameters being dependent variables, whereas the eyes open and closed measurements represented independent variables. Correlation of the results was evaluated with the Spearman rank correlation test at a significance level of *p* ≤ 0.05.

## 3. Results

The research included 55 sedentary females (32 residents of a social care home and 23 patients of a rehabilitation clinic). The age of the participants was 72.60 ± 7.47 (SD). The participants' anthropometrics can be found in Table 1.

Participants with a fall incident in the preceding year formed a faller group. Participants without a fall incident in the preceding year formed a nonfaller group. Fallers were older (*p* < 0.0417) and shorter (*p* < 0.0409) compared to nonfallers. There were no statistically significant differences in body weight, BMI, or WHR between the groups.

A comparative analysis of the results of fallers and nonfallers was conducted. The test of variance showed a difference between the groups in the MLSI EC. One-way ANOVA indicated differences in both groups in TUG (*F*(1.53) = 7.9204), FRT (*F*(1.53) = 11212), MLSI EO (*F*(1.53) = 6.7865), and MLSI EC (*F*(1.53) = 8.4382) values. These results are shown in Table 2.

The Fall Risk Test (BBS) was performed by only 4 participants from the faller group; the remainder were not able to complete the test due to severe disequilibrium. These low numbers precluded statistical testing.

Participants were also divided into two groups, based on their results in the modified Unterberger test. The analysis of variance indicated a difference between the groups in APSI EO and OSI EO. A statistically significant correlation was found between the test and the results of the MLSI EC and OSI EC. The results of the variance analysis are presented in Table 3.

The results of the FRT, TUG, and modified Unterberger test were correlated with stabilographic parameters. The FRT's results do not correlate with the stabilographic parameters. Results of the TUG correlate with the following: OSI EO, MLSI EO, and FRI (instability level 6-2). These results are shown in Table 4.

There is a correlation between the results of the functional tests performed. Participants who had better FRT results also had a shorter TUG time. There is no statistically significant relationship between the modified Unterberger test and other functional tests. These results are shown in Table 5.

## 4. Discussion

Balance is considered to be a complex motor coordination involving various skills. It is based on the interaction of many sensorimotor processes [20]. The increasing elderly population is forcing physiotherapists to attempt to lower the probability and consequences of falling, but this in turn is directly related to appropriate diagnostics [3]. Unfortunately, scientific studies do not provide strong evidence that functional tests are sufficiently reliable to identify FR [21]. This study is aimed at comparing the results of the FRT, TUG, and modified Unterberger test, with stabilographic parameters in FR assessment in older women.

Due to the topic of this study being FR assessment, the study group consisted of women because it is well-known that women are generally more likely to fall than men [3]. Moreover, balance deterioration is associated with old age and postmenopausal oestrogen deficiency [5, 22]. Only women in good mental health were examined. They understood the instructions given, which reduced the risk of incorrect execution of individual tests. Additionally, only women who declared a low level of physical activity during the day were included in the study. Older women have a greater FR and lower physical functional performance [2, 9]. In this research, participants were divided into two groups (fallers and nonfallers). New falls seem to be closely related to fall history [10].

This study shows that the TUG and the modified Unterberger test seem to be the most effective in functional FR assessment in older women. This means that equilibrium disorders can be diagnosed in cheaper and more convenient ways, without a stabilographic platform. The study shows a statistically significant correlation between the results of the TUG, OSI EO, MLSI EO, and FRI6-2, all of which confirms the great effectiveness of the TUG in the functional diagnosis of FR. Furthermore, all these parameters are performed with biofeedback (EO—eyes opened), so the above dependence seems to be very logical. Khan et al. decided to evaluate whether the way the feet are set affects the level of balance. To perform such an evaluation, they used the BBS and TUG. This experiment also confirms the fact that there is a close correlation between the TUG and stabilographic parameter results [23]. Khan et al. concluded that the TUG can be a reliable tool in FR assessment. On the other hand, Brandmeir et al. have shown that TUG is definitely less sensitive in the diagnosis of FR than a stabilographic study on the BBS platform or the Berg scale. The authors suggest that using at least two different functional tests may improve the assessment of FR [24].

Unfortunately, only 4 participants performed the Fall Risk Test (FRI6-2). Two of them were in the FRI value standards for their age range. One reached the lower limit of normal, and the final one exceeded the upper limit. On this basis, it may be concluded that these females experienced a nonbalance-related fall, since they have a well-functioning balance control system. It is possible that in the past, they fell because of external factors. However, 11 participants from the faller group were not able to complete the Fall Risk Test, probably due to an impaired body balance control system. This is confirmed by the research from Oh et al., where they showed a correlation between the OSI, FRI, and TUG values. They correlated the results obtained at various levels of platform instability. They conclude that the balance assessment on the BBS platform should be carried out at lower levels of platform instability. They also prove that TUG is an effective diagnostic tool in fall risk assessment [25].

Moreover, there is a statistically significant difference in OSI EC and MLSI EC between groups with positive and negative results in the modified Unterberger test. Participants with positive results in the modified Unterberger test were characterised by worse stabilographic parameters. During these tests, it is impossible for subjects to compensate for balance deficits by using biofeedback (eyes are closed). These results also confirm the efficacy of the modified Unterberger test in FR assessment.

Melo et al. also find a statistically significant relationship between the results of the Unterberger test and stabilographic parameters. They prove that people with hearing impairment display significantly worse dynamic balance, which may predispose them to a higher FR in the future [17]. Furthermore, Tjernström et al. use the Unterberger test to evaluate the effectiveness of their physiotherapeutic program. They show that, as a result of training, there is a significant reduction in forward movement in the Unterberger test, but there is no statistically significant reduction in the rotation angle [16].

It is worth mentioning that there is no significant correlation between the results of FRT and BBS parameters. This shows that the sensitivity of FRT in FR assessment is low. Movement of the trunk seems to influence the test more than the displacement of the centre of pressure. The FRT does not identify all people with a high FR and, so it is not a sufficiently strong diagnostic tool. All the above results show that FR assessment should take place in a dynamic environment, not in a static one. Jonsson et al. also present no significant correlation between the results of FRT and stabilographic parameters. Therefore, when using the FRT test for assessing balance, compensatory mechanisms should be taken into account [12]. Furthermore, Behrman et al. show that the FRT diagnosed only 30% of patients who previously experienced fall incidents [11]. This shows that the sensitivity of the FRT in FR assessment is low. On the other hand, the results of this study show a correlation between the results of the FRT and TUG. This may indicate that the FRT diagnosed functional decline in older people, but not FR itself. De Waroquier-Leroy et al. prove that the FRT does not seem to be an effective tool to identify FR [13]. They show that joints in the lower limbs and torso are involved during the FRT. Furthermore, worse FRT results were achieved by older women [5, 13]. It should be noted that performing the FRT is only possible because of various strategies and compensations. Wilczyński et al. show a significant relationship between the metabolic age of older people with Parkinson's disease and the values of OSI EO, APSI EO, and the degree of rotation. The parameters of postural stability also deteriorate with age [7].

## 5. Limitations of the Study

Participants declared that their falls could have had different background reasons. It is believed that some of the falls could have been caused by balance disorders (an intrinsic risk factor). The rest of them may have been nonbalance related due to an environmental factor or low levels of physical activity (extrinsic risk factors). On the other hand, participants who did not fall could have shown increased vigilance connected to a fear of falling. Unfortunately, only fifty-five women took part in this study, and further research should be continued on a bigger group of participants.

## 6. Conclusions

The FRT does not seem to be an adequate tool to identify FR, because of the number of compensations used while performing it. However, it can be used to evaluate the functional efficiency of older people. In daily physiotherapeutic practice, the TUG and modified Unterberger test can be used as tools in FR assessment. Indeed, this has been confirmed by the results of the above test with stabilographic parameters. The results indicate that FR assessment should take place in a dynamic environment, not in a static one. In order to improve FR diagnostic accuracy, a physiotherapist should not focus entirely on the results of a single test. Furthermore, the TUG should be performed with an accelerometer placed on the sacrum bone. This would additionally allow deflections in individual plane to be assessed while the subject walks.

## Figures and Tables

**Table 1:** Participants' anthropometrics.

	*n*	Age (years)	Body mass (kg)	Body height (cm)	BMI	WHR
Participants	55	72.60 ± 7.48	70.43 ± 11.33	157.60 ± 6.68	28.43 ± 4.71	0.856 ± 0.092

BMI: body mass index; WHR: waist-hip ratio.

**Table 2:** Results of selected tests divided into groups.

Tests	Fallers			Nonfallers			*p* < 0.05
	*n* = 15			*n* = 40			
	Mini	Max	Mean ± SD	Min	Max	Mean ± SD	
OSI EO	0.20	1.40	0.58 ± 0.29	0.1	1.1	0.44 ± 0.22	0.053
APSI EO	0.1	1.1	0.41 ± 0.26	0.1	0.90	0.35 ± 0.18	0.294
MLSI EO	0.1	0.60	0.31 ± 0.13	0.00	0.70	0.20 ± 0.14	0.0118
OSI EC	0.70	3.10	1.49 ± 0.65	0.50	2.40	1.17 ± 0.52	0.061
APSI EC	0.50	2.10	1.01 ± 0.52	0.30	2.10	0.92 ± 0.45	0.514
MLSI EC	0.20	2.10	0.87 ± 0.57	0.10	1.50	0.51 ± 0.34	0.005
FRT	3.00	38.00	20.9 ± 8.43	13	38	27.7 ± 5.97	0.0015
TUG	5.51	31.5	10.732 ± 7.327	4.98	15.0	7.243 ± 1.874	0.0068

Fallers: participants with a fall incident in the last year; nonfallers: participants without a fall incident in the last year; EO: eyes open; EC: eyes closed; OSI: overall stability index; APSI: anterior-posterior stability index; MLSI: medial-lateral stability index; FRT: Functional Reach Test; TUG: Timed Up and Go.

**Table 3:** Results of stability indices according to the results of modified Unterberger test.

Tests	Group 1			Group 2			*p* < 0.05
	*n* = 32			*n* = 23			
	Min	Max	Mean ± SD	Min	Max	Mean ± SD	
OSI EC	0.50	2.40	1.119 ± 0.525	0.50	3.10	0.488 ± 0.357	0.028
MLSI EC	0.20	1.50	1.456 ± 0.577	0.10	2.10	0.778 ± 0.495	0.014

Group 1: participants with a positive result of the modified Unterberger test; group 2: participants with a negative result of the modified Unterberger test; EC: eyes closed; OSI: overall stability index; MLSI: medial-lateral stability index.

**Table 4:** Values of correlation coefficients of functional tests and stabilographic parameters assessing fall risk.

	Postural Stability Test (PST)						Fall Risk Test
	OSI EO	APSI EO	MLSI EO	OSI EC	APSI EC	MLSI EC	FRI6-2
Functional Reach Test (FRT)	-0.239	-0.086	-0.205	-0.118	-0.143	-0.113	-0.253
Timed Up and Go (TUG)	0.314^∗^	0.231	0.297^∗^	0.104	0.156	0.100	0.435^∗^
Modified Unterberger test	0.206	0.217	0.276^∗^	0.310^∗^	0.169	0.378^∗^	0.097

EO: eyes open; EC: eyes closed; OSI: overall stability index; APSI: anterior-posterior stability index; MLSI: medial-lateral stability index; ^∗^Spearman rank correlation test: *p* < 0.05.

**Table 5:** Values of correlation coefficients of functional tests assessing FRT.

	Functional Reach Test (FRT) (cm)	Timed Up and Go (s)
Timed Up and Go (s)	-0.399^∗^	X
Modified Unterberger test (cm)	-0.112	-0.016

^∗^Spearman rank correlation test: *p* < 0.05.

## Data Availability

The data used to support the findings of this study are available from the corresponding author upon request.

## Additional Points

- The modified Unterberger test TUG seems to be helpful and accurate in FR assessment in older women and can be used to diagnose equilibrium disorders in the absence of a stabilographic platform
- Diagnosing FR should take place in dynamic environments, not in static ones
- Using at least two different functional tests may improve assessment of FR
